# Huntington’s Disease Drug Development: A Phase 3 Pipeline Analysis

**DOI:** 10.3390/ph16111513

**Published:** 2023-10-24

**Authors:** Hannah J. Van de Roovaart, Nguyen Nguyen, Timothy D. Veenstra

**Affiliations:** School of Pharmacy, Cedarville University, Cedarville, OH 45314, USA; hannahjvanderoovaart@cedarville.edu (H.J.V.d.R.); ndnguyen@cedarville.edu (N.N.)

**Keywords:** Huntington’s disease, treatment, clinical trial, phase III, metformin, valbenazine

## Abstract

Huntington’s Disease (HD) is a severely debilitating neurodegenerative disorder in which sufferers exhibit different combinations of movement disorders, dementia, and behavioral or psychiatric abnormalities. The disorder is a result of a trinucleotide repeat expansion mutation that is inherited in an autosomal dominant manner. While there is currently no treatment to alter the course of HD, there are medications that lessen abnormal movement and psychiatric symptoms. ClinicalTrials.gov was searched to identify drugs that are currently in or have completed phase III drug trials for the treatment of HD. The described phase III trials were further limited to interventional studies that were recruiting, active not recruiting, or completed. In addition, all studies must have posted an update within the past year. PubMed was used to gather further information on these interventional studies. Of the nine clinical trials that met these criteria, eight involved the following drugs: metformin, dextromethorphan/quinidine, deutetrabenazine, valbenazine, Cellavita HD, pridopidine, SAGE-718, and RO7234292 (RG6042). Of these drug treatments, four are already FDA approved. This systematic review provides a resource that summarizes the present therapies for treating this devastating condition that are currently in phase III clinical trials in the United States.

## 1. Introduction

Huntington’s Disease (HD) is a rare autosomal dominant neurodegenerative genetic disorder that has an average onset between 30 and 50 years of age [1]. The worldwide prevalence of HD is approximately 7 in 100,000 persons, with further evidence suggesting the worldwide prevalence may be increasing [2,3,4,5,6]. The regions of highest prevalence are North America and the United Kingdom, with Asia having the lowest [4]. This disease places a heavy cost burden on patients, caregivers, and the health system. A recent Huntington’s Disease Burden of Illness (HDBOI) study comprising patients from five European countries and the United States estimated the annual direct medical, nonmedical, and indirect costs associated with HD at just over EUR 63,000 (i.e., about CAD 68,000) per patient per annum [5]. Predictably, these costs escalate as patients progress from early- to late-stage HD. Hospital visits and long-term care account for the largest proportion of these expenses.

Caused by a mutation in the Huntington gene (*HTT*) located on chromosome 4, HD patients have what is known as the “CAG repeat expansion,”. While most healthy people have between 10 and 30 repeats, individuals with HD have 40+ CAG repeats [7]. This trinucleotide repeat inserts additional glutamine residues (referred to as a polyQ domain) within the translated huntingtin protein (HTT). The average age of onset is between 30 and 50 years of age, with individuals possessing longer trinucleotide repeats generally exhibiting symptoms of HD at earlier ages [8]. In predictive models, the length of the trinucleotide repeats accounts for about 70% of the interpatient variability in the age of onset [9]. The highest concentrations of HTT are located within the brain and testes [10]. The main functions of HTT involve chemical signaling, cellular dynamics (i.e., the cytoskeleton, endocytosis, trafficking, and adhesion), metabolism, protein turnover, gene expression (transcription and RNA processing), and protection [11,12]. The aggregation of mutant HTT (mHTT) is the main pathophysiological signature of patients with HD [13]. These aggregates form inclusion bodies inside cells that lead to cell quiescence and neuronal degeneration [14,15]. The clinical manifestations of HD include chorea, dementia, and psychiatric symptoms that eventually lead to death between 15 and 20 years after symptoms are first detected [16,17].

While HD affects the entire brain, there is evidence of enhanced vulnerability within the striatum, which is part of the basal ganglia [18]. This brain region is involved in motor control, emotion, habit formation, and reward [19]. Neuronal degeneration within the basal ganglia is consistent with the symptoms observed in HD patients, which include memory impairment, slurred speech, chorea, weight loss, and personality changes [20,21,22].

Quality of life (QOL) implications are important to assess when considering patients living with HD. A recent review found that factors contributing to a lower QOL included the inability to practice self-care, loss of autonomy with activities of daily living, and increased levels of anxiety and depression [23]. The presence of these effects varies greatly depending on the phase of disease progression, with those in later stages of the disease experiencing a significantly worse QOL. While current disease-modifying therapies are not yet on the market [24], there are several drugs and psychotherapy options available for treating HD symptoms [25]. A complete list of all the currently registered and ongoing clinical trials in HD is periodically published within the Journal of Huntington’s Disease [26]. In addition to this comprehensive list, the articles expand on a couple of trials and provide breaking news on a few others. While these articles are useful, we aimed to provide a detailed summary of HD phase III interventional trials. The hope is that by focusing on this smaller number of trials, investigators will gain a clearer understanding of the strategies and promise of therapies that have already shown efficacy in the treatment of HD.

## 2. Results

ClinicalTrials.gov was used to identify drugs that are currently in or have recently completed a phase III trial for an indication within HD. Only interventional phase 3 trials that were recruiting patients, were active but not recruiting patients, or had been completed were included. All the studies must have had an update posted within the past year.

Nine clinical trials were identified in accordance with the aforementioned criteria. There are eight different drug interventions included in the nine clinical trials. Four of these medications (dextromethorphan/quinidine, valbenazine, deutetrabenazine, and metformin) are FDA approved for alternative indications while four others (Cellavita HD, pridopidine, tominersen, and SAGE-718) have not yet been marketed and are seeking their first FDA approval.

Descriptions of each drug described are shown in the following manner: (1) background information about the drug; (2) overview of evidence supporting the use of the drug in HD; and (3) an overview of the current phase III trial. While most of the information concerning the clinical trial was obtained from the Clinicaltrials.gov website, PubMed was used to identify additional studies that provide additional information on the clinical trial and substantiate the testing of the drug for treating HD. The nine drugs described in this report, along with some brief clinical trial information, are listed in Table 1.

### 2.1. Metformin

Metformin is one of the oldest and most studied diabetic medications [27,28]. Besides type 2 diabetes (DM2), metformin may also offer cardiovascular protection and beneficial effects on obesity, musculoskeletal and reproductive diseases, cancer, and aging [29]. Not only is metformin able to penetrate nearly every bodily organ, but it is also a well-known pleiotropic agent that modulates a plethora of metabolic pathways, extending its possible use beyond already FDA-approved indications [30]. The major molecular targets of metformin include complex I of the mitochondrial electron transport chain, the mechanistic target of rapamycin complex 1 (mTORC1), and adenosine monophosphate-activated protein kinase (AMPK) [31]. The interest in metformin as an HD treatment is in its ability to activate AMPK. Located throughout the body, this enzyme induces improved neuronal survival by inhibiting inflammation and promoting cell renewal processes [32]. Metformin has also been shown to induce autophagy, inhibit mHTT aggregation, and reduce the accumulation of this mutant protein within an HD mouse model [33]. While the metformin activation of AMPK may offer some benefits, the optimal timing of AMPK activation appears to be an important variable in its effectiveness as an HD therapeutic. Data from in vivo/in vitro models and analyses of brain tissue have demonstrated that AMPK activation during late stages of HD could have negative effects [34]. Thus, AMPK may be an efficacious target in early-stage HD but may have contrasting effects during late-stage HD. 

#### 2.1.1. Overview of Evidence Supporting Use of Metformin in HD

There is evidence supporting the correlation between HD and metabolic dysregulation, potentiating the impact of metabolic modulators in the treatment of HD [35,36,37]. This dysregulation affects the metabolism of tricarboxylic acids, urea, amino acids, and lactate [38]. Several genes associated with metabolism including glucose transporter 1 (GLUT1), GLUT3, and insulin along with members of the sortilin family [39,40,41] have been shown to be altered in HD. Considering the important effects of metformin on insulin sensitivity and overall metabolic health, investigators have studied this drug as a therapy to help individuals with HD who suffer from metabolic dysregulation.

#### 2.1.2. Overview of Current Phase III Trial

A phase 3 clinical trial titled “Testing METformin against cognitive decline in HD” was approved to test the efficacy and safety of treating HD patients with metformin at a dose of 1700 mg/day “https://classic.clinicaltrials.gov/ct2/show/NCT04826692 (accessed 12 September 2023)”. This randomized, double-blind, placebo-controlled study divides 60 patients equally into experimental and placebo comparator groups, with the experimental group being given 425 mg of metformin twice a day for 2–4 weeks, and if they tolerate this dose well, 850 mg of the drug twice a day for 52 weeks. The estimated study completion date is August of 2024. 

This study’s primary outcome is the evaluation of the drug’s effect on scores obtained in different cognitive subtests within the Unified Huntington’s Disease Rating Scale (UHDRS) from baseline to 52 weeks [42]. The three primary subtests being evaluated include (1) the verbal fluency test under a phonetic slogan with the letters F, A, and S, aiming to assess verbal fluency; (2) the words and interference in the Stroop test, which consist of color words printed in different colored ink assessing participants’ ability to recognize varying stimuli among others to assess selective attention and inhibitory response; and (3) the Symbol Digit Modalities Test [43] that aims to assess psychomotor speed by providing symbol–digit pairs alongside symbols and asking patients to match as many symbols to their corresponding number as possible.

A previous post hoc analysis of the Enroll-HD database, a world-wide observational study of 7000 patients, analyzed data to identify differences between HD motor manifest patients who take metformin and those who do not. While metformin takers tended to score better on the UHDRS test, the result did not reach statistical significance (*p* = 0.09) [34]. Similarly, the Stroop color naming test and the Stroop word reading test showed strong trends in favor of those taking metformin (*p* = 0.058 and *p* = 0.053, respectively). Statistical significance was found, however, for the overall UHDRS cognitive score (*p* = 0.003), verbal fluency test (*p* = 0.004), and the Stroop interference test (*p* < 0.001). 

So, why is the phase 3 trial described above important? The patients in the Enroll-HD study taking metformin were all diabetic. Diabetes is known to negatively affect cognitive test scores [44]. Properly evaluating an association between metformin use and cognitive function among HD patients requires comparing HD patients taking metformin against HD patients who do not. The “Testing METformin against cognitive decline in HD” trial is specifically designed to evaluate these two groups.

### 2.2. Dextromethorphan/Quinidine

Dextromethorphan/quinidine (DM/Q) is a fixed-dose combination therapy that was approved by the FDA in 2010 [45]. It is marketed by Avanir Pharmaceuticals under the brand name Nuedexta. Dextromethorphan is a noncompetitive N-methyl-D-aspartate (NMDA) receptor antagonist and a sigma-1-receptor (S1R) agonist [46]. Quinidine prolongs the plasma levels of dextromethorphan by inhibiting the CYP2D6 enzyme. There is evidence that NMDA receptor excitotoxicity is implicated in the pathogenesis of HD. Enhanced NMDA receptor signaling is detected at ages prior to motor dysfunction and neuronal loss. Specifically, agents such as quinolinic acid (a selective NMDA receptor agonist) can produce striatal degeneration. There are studies in HD mice treated with NMDA receptor antagonists that showed the reversal of nuclear signaling and motor learning deficits. Pridopidine, an S1R agonist, has previously been shown to have neuroprotective effects in cellular and animal models of HD (as well as AD) presumably by increasing mitochondrial activity. This increased activity led to improvements in motor coordination and a delay in symptom onset in mouse HD models.

#### 2.2.1. Overview of Evidence Supporting Use of Dextromethorphan/Quinidine in HD

DM/Q is prescribed for treating pseudobulbar affect (PBA), also known as emotional lability, labile affect, or emotional incontinence [47]. Individuals with PBA involuntarily cry or laugh even in the absence of any event that would normally trigger such a response [47]. These symptoms can be severe, persistent, and occur unpredictably. These uncontrolled outbursts can last anywhere from a few seconds to several minutes and occur several times per day. PBA is often observed in patients with Alzheimer’s Disease (AD), amyotrophic lateral sclerosis (ALS), multiple sclerosis (MS), Parkinson’s Disease (PD), and traumatic brain injury (TBI) [48]. While not commonly reported in patients with HD, there is at least one report of a patient with late onset HD exhibiting signs of PBA [49].

#### 2.2.2. Overview of Current Phase III Trial

Based on the FDA’s approval of DM/Q for treating PBA, investigators at The University of Texas Health Science Center in Houston, TX, initiated a phase 3 interventional trial entitled “Evaluating the Efficacy of Dextromethorphan/Quinidine in Treating Irritability in Huntington’s Disease”. This study of 20 individuals is designed to assess the efficacy and safety of DM/Q 20 mg/10 mg in HD patients who exhibit irritability. Only verified HD mutation carriers with a baseline irritability score > 14 were included. Patients also had to have not changed their medication for 30 days prior to the DM/Q or placebo treatment. The study consists of two participant groups: experimental and placebo comparator. The experimental group receives one DM/Q 20 mg/10 mg capsule daily for 1 week, followed by DM/Q 20 mg/10 mg twice daily for 4 weeks and finally DM/Q 20 mg/10 mg once daily for 7 days. The placebo comparative arm is given the same dosing schedule; however, they receive a placebo in place of DM/Q 20 mg/10 mg.

The primary outcomes of the study are to measure changes in patient’s irritability as assessed by The Irritability Scale, which ranges from 0 (not irritable) to 42 (highly irritable). Patients’ irritability was assessed at baseline and at weeks 6 and 13 of treatment. Secondary outcomes included behavioral symptoms measured by using tools such as the Neuropsychiatric Inventory Questionnaire (NPI-Q) and Problems Behavior Assessment (PBA), motor symptoms as measured via a total motor score (TMS) and total maximal chorea (TMC), cognitive symptoms as measured by using the Montreal Cognitive Assessment (MoCA) test, functional independence as assessed by using the UHDRS Total Functional Capacity (TFC) scale, and patient progress and treatment as observed by a clinician using the Clinical Global Impressions Scale (CGI). The actual study completion date is listed as 11 November 2022; however, no results have been posted.

### 2.3. Deutetrabenazine

As a reversible inhibitor of vesicular monoamine transporter 2 (VMAT2), deutetrabenazine decreases the reuptake and stores monoamines (i.e., dopamine, serotonin, histamine, and norepinephrine) within presynaptic vesicles [50]. VMAT2 is located on the synaptic vesicles of presynaptic neurons [51]. Responsible for the packaging and release of monoamines into the synapse, drugs targeting this transporter have shown efficacy in treating symptoms that manifest from the imbalance of monoamines [52]. 

Deutetrabenazine is a deuterated form of tetrabenazine, meaning that there is a substitution with deuterium for hydrogen throughout the molecule, giving it a plasma half-life (t½) almost two-fold greater than tetrabenazine [53]. This longer t½ decreases plasma fluctuations, resulting in fewer adverse effects such as somnolence, insomnia, depression, and Parkinsonism, which are associated with peak drug concentration.

#### 2.3.1. Overview of Evidence Supporting Use of Deutetrabenazine in HD

Tetrabenazine was approved by the FDA in 2008 and has been the standard of care for the treatment of HD-associated chorea for nearly a decade [54]. Analyses of large-scale phase III studies suggest that while efficacy outcomes are similar between the tetrabenazine and deutetrabenazine agents, the tolerability profile strongly favors deutetrabenazine [53]. While important to note, a true comparison between these agents cannot be evaluated until a focused head-to-head study is conducted.

First-HD was a phase III trial that evaluated the efficacy and safety of deutetrabenazine for controlling HD-associated chorea [55]. Ninety HD patients with maximal chorea scores equal to or greater than eight (a higher score indicates more chorea) were randomized to receive deutetrabenazine (*n* = 45) or a placebo in a double-blinded fashion. The change in the total maximal chorea score was used as the endpoint. Within 12 weeks, those given deutetrabenazine showed an improvement in the mean maximal chorea score from 12.1 to 7.7, while the improvement in the placebo group went from 13.2 to 11.3. Furthermore, after the 1-week washout period, chorea scores returned to baseline in the treatment group, reinforcing the positive impact of the treatment. This study led the FDA to approve deutetrabenazine for chorea associated with HD and tardive dyskinesia (TD) in 2017 [56].

#### 2.3.2. Overview of Current Phase III Trial

The phase 2/3 trial entitled “Impact of Deutetrabenazine on Functional Speech and Gait Dynamics in Huntington Disease” is currently evaluating the effect of this drug on the functional speech and gait dynamics in HD [57]. With 30 participants and an open-label single-arm design, the primary outcome measures of this study are (1) the Sentence Intelligibility Test (SIT) and (2) Motor Speech Evaluation (MSE) [58]. The SIT measures speech intelligibility by requiring each participant to read aloud 11 sentences that increase in length from 5 to 10 words. The SIT score is measured by dividing the mean number of correct words transcribed from the sentences by three unfamiliar listeners by the total number of words in the 11 sentences. The MSA includes a series of tasks (i.e., vowel prolongation, alternating motion rates, sequential motion rates, and contextual speech) that enable a consistent rating of characteristics perceptible within motor speech disorders. All participants will be given oral deutetrabenazine for a duration of 2 years starting at a dose of 6 mg/day and uptitrated to a max dose of 48 mg/day [57]. This trial has an estimated completion date of April 2024.

### 2.4. Valbenazine

Hyperkinetic movements including those representative of chorea have been associated with high dopamine states. Thus, the current standard of care aims to decrease dopamine activity through the off-label use of antipsychotics that antagonize postsynaptic receptors or the on-label use of the existing FDA-approved VMAT2 inhibitors deutetrabenazine and tetrabenazine [59]. As a VMAT2 inhibitor, valbenazine works similarly to deutetrabenazine and tetrabenazine to treat chorea associated with HD. Valbenazine differs from deutetrabenazine and tetrabenazine in its affinity for VMAT2. Deutetrabenazine and tetrabenazine are broken down into four different stereoisomers, all of which have shown varying affinity for VMAT2. Valbenazine is broken down into only one stereoisomer, [+]-α-dihydrotetrabenazine, which has the strongest affinity for VMAT2 of all the stereoisomers [59]. In addition to the affinity observed with respect to VMAT2 receptors, valbenazine’s formulation allows for easier titration and daily dosing. Deutetrabenazine and tetrabenazine require multiple episodes of daily dosing, with tetrabenazine specifically requiring a slower, more complicated uptitration schedule (package inserts). 

#### 2.4.1. Overview of Evidence Supporting Use of Valbenazine in HD

**Figure 1:**
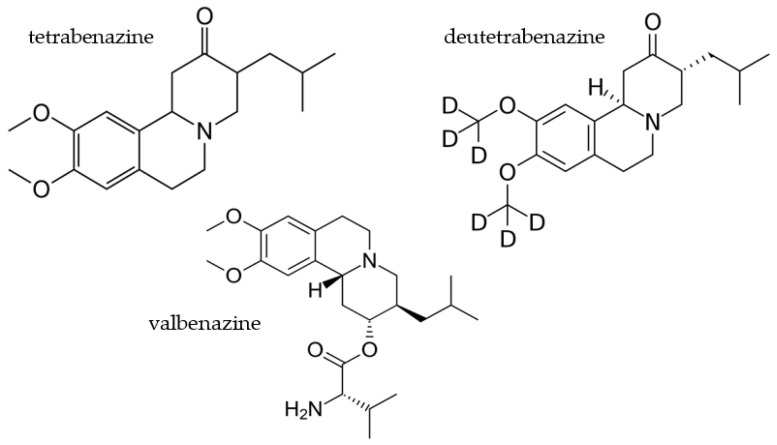
Comparison of VMAT2 inhibitors tetrabenazine, deutetrabenazine, and valbenazine.

While not identical, TD and chorea both involve involuntary movements that affect various parts of the body [60]. While TD primarily involves the oral–buccal–lingual region, chorea involves the arms and legs, which are parts of the body that affect the gait and daily functions of the patient. Chorea is classified as a type of dyskinesia. There are many factors that justify testing valbenazine as an agent for treating HD-associated chorea. Firstly, valbenazine was approved by the FDA as the first treatment for TD in 2017 [61]. Considering the similarities between chorea and TD, it seems logical to test the efficacy of treating HD-associated chorea with valbenazine. Secondly, tetrabenazine has been approved for the treatment of HD-associated chorea since 2008 [62]. Thirdly, deutetrabenazine has shown positive results in lessening the symptoms of chorea in HD patients [63]. Finally, valbenazine, tetrabenazine, and deutetrabenazine have similar structures (Figure 1), and their mechanisms of action all involve acting as VMAT2 inhibitors.

#### 2.4.2. Overview of Phase III Clinical Trials

Valbenazine is currently undergoing studies for an FDA indication of chorea associated with HD. Neurocrine Biosciences, in collaboration with the Huntington Study Group, recently completed their KINECT-HD phase III randomized, double-blind, placebo-controlled trial that evaluated the efficacy and safety of valbenazine to treat chorea in patients with HD. In this study entitled “Efficacy, Safety, and Tolerability of Valbenazine for the Treatment of Chorea Associated with Huntington Disease (KINECT-HD)”, an experimental group received valbenazine in capsule formulation once daily for 12 weeks, with the placebo group receiving a comparator capsule [59]. The primary outcome was a change in the UHDRS TMC score from baseline to the maintenance period (an average of weeks 10 and 12 scores). A decreased score indicates an improvement in chorea. While KINECT-HD wrapped up in late 2021, there was a certification of extension submitted in September of 2022, granting the investigators an additional 2 years before submitting any results. Results, however, were recently published in Lancet Neurology with positive findings for valbenazine use in HD patients with chorea. Of the 125 participants included in the full analysis set, the change in the UHDRS TMC score from baseline to maintenance was −1.4 in the placebo and −4.6 in the valbenazine groups (*p* < 0.0001). An overall improvement in chorea severity was observed by clinicians and patients as early as 2 weeks after the initial dose. The most reported significant treatment-emergent adverse event (TEAE) was somnolence, with 10 patients in the treatment group and 2 in the placebo group suffering excessive sleepiness [59]. 

An ongoing phase III extension of the KINECT-HD study entitled “An Open-Label Rollover Study for Continuing Valbenazine Administration for the Treatment of Chorea Associated With Huntington Disease” is currently examining the long-term safety and tolerability of valbenazine as its primary outcome measure [64]. Its secondary outcome measure is a change from the baseline in the UHDRS TMC score up to 156 weeks. The participants in this study (n = 150) must have either (1) completed study NBI-98854-HD3005, (2) participated but terminated dosing early due to COVID-19 site disruptions, or (3) have a clinical and genetic diagnosis of HD with chorea and be able to walk with or without the assistance of a person or device. With the primary outcome of measuring TEAEs up to 156 weeks, this study’s estimated completion date is December of 2023.

### 2.5. Cellavita HD

Cellavita HD represents a novel therapy within the treatment landscape for HD. Cellavita HD is a stem cell therapy that may help restore lost brain cells [65]. Cellavita HD involves dental-pulp-derived mesenchymal stem cells (DMSCs) that have been associated with both multifaceted differential effects as well as immunomodulatory functions [66]. Capable of crossing the blood–brain barrier (BBB), Cellavita HD promotes the proliferation of neuronal stem cells. Available evidence supporting the use of dental pulp stem cells (DPSCs) in neurodegenerative disorders comes from their ability to regulate different molecular mediators including those within the anti-inflammatory, neurogenic, antiapoptotic, angiogenic, and osteogenic classes [67].

#### 2.5.1. Overview of Evidence Supporting Use of Cellavita HD in HD

The clinical utility of DMSCs varies greatly and includes, but is not limited to, tissue engineering, tooth regeneration, and the treatment of relevance degenerative diseases [68]. In vivo data found that the administration of stem cells in HD increased brain-derived neurotrophic factor (BDNF) levels in the striatum and cortex of the brain, resulting in enhanced neuroprotection [69]. DMSCs have the capacity to differentiate into neuronal and glial cells, two types of cells that are either lost or lose their normal functions in patients with HD [70]. With their capacity for neural repair and neurogenesis, along with their neuroprotective properties, DMSCs represent a potentially useful treatment for neural tissue regeneration in the brains of individuals suffering with HD.

#### 2.5.2. Overview of Phase III Clinical Trial

This phase II/III trial evaluating Cellavita HD entitled “Clinical Extension Study for Safety and Efficacy Evaluation of the Cellavita HD Administration in Huntington’s Patients (ADORE-EXT)” is an extension of the phase II dose-response ADORE-DH study [71]. Participants will receive the maximum dose tested in the prior study, receiving a total of twelve intravenous administrations dosed at 2 × 10^6^ cells/weight range divided into three administrations per cycle. Each infusion occurs every 30 days and cycles every 180 days with a total of four cycles. As a single-group, open-label treatment study, the primary endpoint was the maintenance of therapy effectiveness regarding clinical progression during the 2-year study period. The maintenance of effectiveness is being evaluated by comparing the total UHDRS score registered at baseline and then again at the 2-year mark. Total enrollment for this study is on the lower end for a phase III trial with only 35 participants. With the Cellavita HD trials occurring exclusively in Brazil, the available pool of participants is limited by the single center design and subsequent lack of geographic reach.

### 2.6. Pridopidine

In HD, there is progressive neuronal loss in the striatum. This process includes the dysregulation of endoplasmic reticulum (ER) calcium, altered mitochondrial function, reduced autophagy, increased ER stress, and reduced BDNF, all ultimately leading to neuronal death [72]. Pridopidine was originally identified as a low-affinity dopamine D2 receptor ligand that modulated dopamine-dependent behaviors [73]. In the R6/2 mouse model of HD, pridopidine had been shown to improve motor performance and offer neuroprotective effects [74]. Recent evidence, however, has shown that pridopidine’s affinity for S1R is ~100- and ~30-fold higher than that for the D2 and D3 receptors, respectively [73]. These results suggest that the positive effects of pridopidine are modulated through its interaction with S1R. Studies have shown that pridopidine activation of S1R reduces cell stress and inflammation, while increasing cellular energy production and the clearance of toxic proteins. Since S1R is also involved in synapse plasticity, pridopidine can enhance neuronal connectivity. Prilenia Pharma currently holds orphan drug designation for the treatment of HD in both the US and EU using pridopidine, while simultaneously receiving fast-track designation from the FDA [75].

#### 2.6.1. Overview of Evidence Supporting Use of Pridopidine in HD

Pridopidine exerts its efficacy through the neuroprotective S1R, which is expressed at very high levels in the brain. Crossing the BBB and agonizing S1R frees up this protein, allowing it to interact with numerous other targets. Some of the downstream effects include removing toxic proteins from the brain, enhancing BDNF secretion, protecting neurons from damage, and regulating calcium flow [75]. In vitro and in vivo clinical data have demonstrated pridopidine’s S1R-mediated protective mechanisms from mHTT [76]. Most notable was the positive effect that pridopidine treatment had on TFC throughout the PRIDE-HD trial. Furthermore, this improvement was maintained over an additional 4 years when participants were followed during the open-label study [77].

Initially aiming to evaluate pridopidine’s symptomatic benefit with regard to movement improvement, the focus of the PRIDE-HD trial shifted after the results yielded a negative result using this primary endpoint. A post hoc evaluation found that while no motor improvement was seen, TFC was better maintained in HD patients taking pridopidine [77]. This measure of improvement in UHDRS TFC showed that pridopidine slowed the progression of neuronal loss and suggested the drug could subsequently be useful as a disease-modifying therapy [78].

#### 2.6.2. Overview of Phase III Clinical Trial

Pridopidine has recently completed a phase III trial evaluating the safety and efficacy of pridopidine given 45 mg twice daily to patients with early-stage manifest HD. Known as PROOF-HD (PRidopidine’s Outcome On Function in Huntington Disease), this study is a randomized, parallel, double-blind, placebo-controlled trial with nearly 500 participants [79]. Study locations spanned several countries including Austria, Canada, Czechia, France, Germany, Italy, Netherlands, Poland, Spain, the United States, and the UK. Divided into two treatment arms, the experimental group received a 45 mg hard gelatin capsule twice daily, and the placebo comparator received a matching hard gelatin placebo capsule twice daily. The primary outcome evaluated the change in the UHDRS-TFC score from baseline to 65 weeks postrandomization. The patients who completed this main study will be eligible for the open-label extension. The study completion date is listed as April 7, 2023; however, no results have yet been posted. 

### 2.7. SAGE-718

After receiving orphan drug status and fast-track designation for the treatment of HD by the European Medicine Agency, SAGE-718 has gained traction in both its clinical development and regulatory review process. As a first-in-class NMDA receptor modulator, SAGE-718 was designed to improve cognitive function in disorders associated with NMDA dysfunction such as AD and HD [80]. Modulation of the NMDA receptor enhances long-term effects at neuronal synapses, which is an essential process in learning and memory. 

#### 2.7.1. Overview of Evidence Supporting Use of SAGE-718 in HD

**Figure 2:**
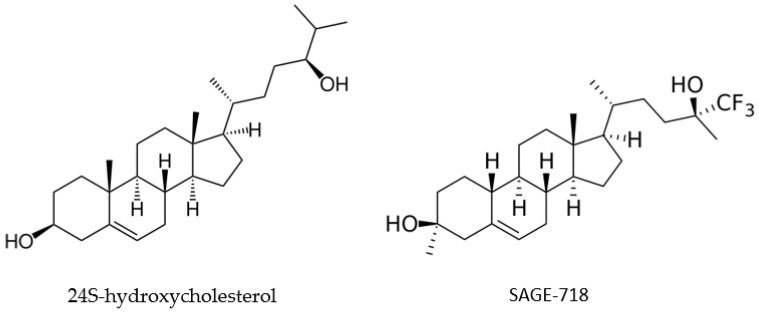
Comparison of 24S-hydroxycholesterol and SAGE-718 structures.

Finding evidence to support SAGE-718′s potential effectiveness in treating HD requires examining 24S-hydroxycholesterol. In a biomarker discovery study of 96 HD patients and 62 controls, 24S-hydroxycholesterol levels were found to be significantly lower in HD-affected individuals compared to controls (*p* < 0.001) [81]. In addition, the decline in plasma 24S-hydroxycholesterol levels paralleled the patients’ transition from premanifest HD to stage 1, which is characterized by neuronal loss and motor disease. An additional observational study found that 24S-hydroxycholesterol levels decreased as patients presented with more progressive stages of HD [82]. This decrease may result in NDMA hypofunction since this steroid serves as an endogenous modulator of NDMA receptors. The activation of these receptors results in the transmission of excitatory signals that influence cognition and other brain functions. Owing to its structure being similar to 24S-hydroxycholesterol, SAGE-718 is hypothesized to bind to the NDMA receptor, restoring its activity and potentially improving cognition in HD patients (Figure 2).

#### 2.7.2. Overview of Phase III Clinical Trial

“A Study to Evaluate the Safety and Tolerability of SAGE-718 in Participants With Huntington’s Disease” aims to recruit 300 participants for a multicenter, open-label study to evaluate the long-term safety and tolerability of SAGE-718 in patients with HD. The study’s estimated completion date is December 2025. Three arms are included: cohort 1: participants directly rolled over from the 718-CIH-210 study; cohort 2: participants with a gap of >7 days since the 718-CIH-201 study; and cohort 3: participants who were not previously enrolled in any SAGE-718 study. All three cohorts will be dosed with an oral softgel lipid capsule of SAGE-718 from day 1 up to day 365. Participants must be between the ages of 25 to 65 years and have CAG expansions of at least 40 trinucleotide repeats. 

The four primary outcomes being evaluated in this study are (1) the percentage of participants with TEAEs and the severity of TEAEs; (2) the number of participants who withdraw due to adverse events (AEs); (3) the percentage of participants with a change from baseline in vital signs, clinical laboratory parameters, and electrocardiograms parameters; and (4) a change from baseline in the Columbia Suicide Severity Rating Scale (C-SSRS) responses. 

### 2.8. Tominersen (RO7234292 or RG6042)

**Figure 3:**
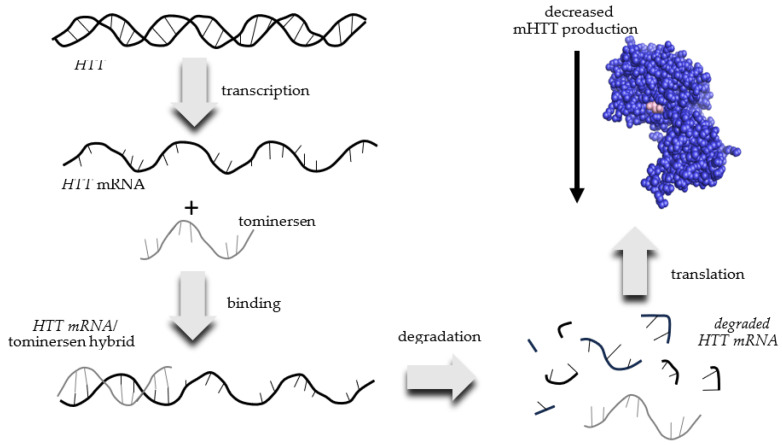
Mechanism of action of tominersen for the decreased production of mutant huntingtin (mHTT) protein in patients with Huntington’s Disease.

Tominersen is also known as RO7234292 or RG6042. Ionis Pharmaceuticals designed tominersen and licensed the treatment to Roche, who is currently developing and marketing it as a potential HD treatment. Tominersen is an antisense RNA that binds to mRNA transcribed from the mutant *HTT* gene found in HD patients. This interaction creates an *HTT* mRNA/tominersen hybrid, which is degraded by the cell and is therefore not translated into the mHTT protein (Figure 3). The net result is a reduced production of mHTT.

#### 2.8.1. Overview of Evidence Supporting Use of Tominersen for HD

Being an autosomal dominant disorder with a single, well-known genetic target would seem to make HD an ideal candidate for gene therapy [83]. The primary goal of gene therapies for neurodegenerative conditions is to reduce gene expression at either the transcriptional or translational level. While clustered regularly interspaced short palindromic repeats (CRISPR) technology holds great potential for gene editing, it has shown some limitations, including having a low on-target editing efficiency, incomplete editing, and inaccurate on- and off-target editing [84]. Interrupting protein production at the mRNA level by using antisense oligonucleotides (ASOs) has been employed previously for the treatment of neurodegenerative conditions. In fact, eteplirsen and inotersen have been approved by the FDA for the treatment of Duchenne muscular dystrophy and familial amyloid polyneuropathy, respectively [85]. A phase 1/2 trial (NCT02519036) conducted previously showed a significant reduction in mHTT levels within the cerebrospinal fluid in patients treated with tominersen [86,87]. These results generated hope that tominersen could reduce the mHTT burden within the neurons of HD patients and slow disease progression.

#### 2.8.2. Overview of Phase III Clinical Trial

Unfortunately, the previous phase 1/2 study was discontinued in phase 3 as it generated a poor risk–benefit profile. The patients who were given tominersen intrathecally every 8 weeks showed worse composite UHDRS scores than those given the placebo. The patients dosed with tominersen or placebo every 16 weeks had comparable composite UHDRS scores. While this study was discontinued, another phase 3 study entitled “An Open-Label Extension Study to Evaluate Long-Term Safety and Tolerability of RO7234292 (RG6042) in Huntington’s Disease Participants Who Participated in Prior Roche and Genentech Sponsored Studies” was initiated with three primary outcomes. Firstly, the study will measure the percentage of participants who develop TEAEs that arise up to 5 months after the last study’s drug intake. Secondly, patterns of suicidal thoughts and behaviors are going to be monitored. The C-SSRS will be used to record the number of participants that have suicidal ideations and exhibit suicidal and self-injurious behaviors without the intent of suicide. Thirdly, the efficacy of the treatment will be assessed by measuring a change in cognition from baseline by using the Montreal Cognitive Assessment (MoCA). The MoCA measures a series of basic assessments, including short-term and working memory, attention, concentration visuospatial abilities, language, and orientation to time and place. These outcomes will be assessed up to approximately three years after the treatment. While the results of this study have not yet been published in a peer-reviewed journal, the results listed on Clinicaltrials.gov do not suggest a significant change in MoCA scores in patients treated every 8, 12, or 16 weeks.

## 3. Discussion

There is currently a wide array of therapeutic modalities being studied to treat the symptoms and underlying cause of HD. Two VMAT2 inhibitors, deutetrabenazine and valbenazine, are currently being tested for their ability to improve functional speech/gait dynamics and reduce chorea, respectively. VMAT2 inhibitors have shown promising results in previous research studies for the symptomatic management of HD. While both deutetrabenazine and valbenazine can reduce symptoms, they do not halt or slow HD progression. Other drugs such as SAGE-178 can also reduce cognitive decline by restoring NDMA function. Unfortunately, these treatments do little to reduce HD progression.

To at least slow disease progression requires interventions that reduce neuronal loss or replenish lost neurons. Metformin, a well-known type 2 diabetes mellitus drug, has shown a positive effect in animal models of HD [88]. However, this drug has also been associated with adverse outcomes and cognitive impairment in animal models of Alzheimer’s and Parkinson’s Disease. Whether metformin’s ability to protect neurons through attenuating AMPK activation improves cognitive function in humans may be answered by current clinical trials. Another drug target being explored to reduce HD burden is S1R NMDA receptors. By preferentially binding to these receptors, pridopidine can reduce cellular stress and inflammation while increasing energy and toxic protein clearance, which provides hope that the loss in the neuronal connectivities within the brains of HD patients can be slowed. Cellavita HD represents potentially the most hopeful mechanism for slowing HD progression. This treatment does not attempt to intervene on already damaged neurons but employs DMSCs to replace damaged cells. With their ability to differentiate into both neuronal and glial cells, these mesenchymal stem cells have the potential to regenerate lost neural tissue in HD patients. The efficiency and tolerability of Cellavita HD will dictate whether (and to what extent) HD progression is slowed or symptoms are mitigated.

While the drugs mentioned above treat symptoms related to HD or slow its progression, none are a cure. Considering the immense physical, mental, and emotional challenges individuals and their caregivers face during the course of this disease, elongating this period can seem like little consolation. To truly alleviate the suffering perpetuated by any neurodegenerative disease requires nothing less than a cure. For HD, a curative agent would eliminate the expression of *mHTT* so that only the wild-type HTT allele is expressed. That is the express aim of tominersen. This ASO treatment binds to *mHTT* mRNA, preventing it from being translated to mHTT protein. Removing the mutant protein responsible for forming aggregates eliminates the neuronal death observed in HD patients.

While some of the drugs described above slow the progression of symptoms, patients with HD will ultimately struggle to meet their nutritional needs, and they typically have less than normal body weights for their height [89]. Whether this deficiency is due to chorea, metabolic changes, or other factors remains to be determined, but HD patients have higher-than-average calorie needs [90]. Patients with HD may need to change their eating habits to meet this need [91]. For example, eating smaller, more frequent meals; avoiding beverages without calories or drinking between meals; and using cues to indicate eating times may help increase caloric consumption. As the disease progresses, mechanical devices such as large-handled utensils, soup plates, heated dishes, and sports cups can make food and beverage consumption easier and more palatable for HD patients. Food preparation devices such as blenders, processors, and juicers can puree difficult-to-chew foods such as fruits and vegetables that provide much-needed nutrients. Besides using mechanical means to maintain food intake, it is also important for HD patients to increase their caloric intake without having to increase the total amount of food they eat. Calories can be increased simply by adding things such as sauces or sugar to various foods, using whole milk rather than skim milk, using high-calorie spreads (i.e., peanut butter, mayonnaise, cream cheese, etc.), or supplementing their diet with high-calorie nutritional drinks [e.g., BOOST^®^ (Vevey, Switzerland), Ensure^®^, (Abbott Park, IL, USA), etc.].

Having no cure, it is important for individuals at risk for developing HD to develop dietary habits that may delay neurological conditions such as dementia. Some of these “brain healthy” habits include a diet rich in antioxidants, anti-inflammatories, and vitamin B12 [92]. Recommended foods with antioxidant properties include fruits and vegetables, which may need to be pureed to aid consumption. Omega 3 fatty acids in foods such as olive oil, avocados, and nut butters can help reduce inflammation while reducing triglyceride levels. Vitamin B12, which is found in meats, dairy, eggs, and poultry, can help preserve brain and nerve function. Obviously, the health benefits provided by these foods are not exclusive to individuals with or at risk for HD. Unfortunately, many late-stage HD patients are no longer able to take in adequate nutrients by mouth and must resort to a feeding tube [93]. Nutrition can be administered either continuously, intermittently, as a bolus, or as a combination of intermittent administration and a bolus.

As with most diseases, genome-wide association studies (GWASs) have also been applied to globally identify genetic variations with HD patients. A recent GWAS of 6000 to 9000 HD patients identified several genes involved in DNA-damage repair as significant modulators of age at onset and disease severity [94,95]. Many of these disease-modifying DNA-repair genes are involved in mismatch repair (MMR), base-excision repair (BER), or nucleotide salvaging [96]. Another hallmark of HD is an increase in the reactive oxygen species (ROS)—a feature common to many late-age-onset neurological diseases [97]. Considering that ROS are known to cause significant DNA damage, it is logical to assume that deficiencies in DNA-repair enzymes exacerbate HD symptoms and progression. These recent findings implicating DNA-repair enzymes in HD progression present new therapeutic opportunities. Unlike cancer, where the aim is to eliminate affected cells, therapeutic strategies in neurological diseases are used to save affected cells or fortify target cells. While lifestyle behaviors can aid in the reduction in ROS and enhance DNA repair to a degree, the goal would be to generate a pharmacological agent to stimulate DNA repair. While there are several agents that induce DNA damage, there are few that act to enhance DNA repair. A recent report described enoxacin, a molecule that augments endoribonuclease DICER activity resulting in a stronger DNA damage response and repair [98]. While this molecule stimulates the repair of double-strand breaks, it shows the potential of developing pharmacological agents that may stimulate MMR or BER enzymes to enhance the survival of cells affected by HD.

## 4. Conclusions

Promising symptomatic and disease-modifying therapies are making their way through clinical trials for the treatment of HD. These therapies include addressing metabolic dysregulation with metformin, accounting for the emotional lability and irritability with dextromethorphan/quinidine, treating symptomatic movement disorders with valbenazine and deutetrabenazine, slowing the progressive loss of neurons with pridopidine, improving cognition via NMDA modulation with SAGE-718, and even promoting neurogenesis with stem cell therapy. While curing HD would be the ultimate prize, any treatment that can slow its progression and allow patients to live a normal life would be a tremendous advancement.

## Figures and Tables

**Table 1:** List of phase III clinical trials testing the effects of various drugs in the treatment of Huntington’s Disease.

Drug	Clinical Trial Name	Identifier	Primary Outcome
Metformin	Testing METformin against cognitive decline in HD	NCT04826692	Evaluate drug’s effect on scores obtained in different cognitive subtests that make up the Unified Huntington’s Disease Rating Scale (UHDRS)
Dextromethorphan/ quinidine	Evaluating the Efficacy of Dextromethorphan/Quinidine in Treating Irritability in HD	NCT03854019	Measure patient’s irritability using The Irritability Scale
Deutetrabenazine	Impact of Deutetrabenazine on Functional Speech and Gait Dynamics in Huntington Disease	NCT04713982	Improvement on Sentence Intelligibility Test and Motor Speech Evaluation
Valbenazine	Efficacy, Safety, and Tolerability of Valbenazine for the Treatment of Chorea Associated with Huntington Disease (KINECT-HD)	NCT04102579	Improvement in chorea symptoms and evaluation of treatment-emergent adverse events
	An Open-Label Rollover Study for Continuing Valbenazine Administration for the Treatment of Chorea Associated With Huntington Disease	NCT04400331	Evaluation of treatment-emergent adverse events (TEAEs)
Cellavita HD	Clinical Extension Study for Safety and Efficacy Evaluation of the Cellavita HD Product in Huntington’s Patients (ADORE-EXT)	NCT04219241	Evaluate treatment’s effectiveness as verified by comparing the total UHDRS
Pridopidine	Pridopidine’s Outcome on Function in Huntington Disease, PROOF-HD	NCT04556656	Measure change from baseline in UHDRS-Total Functional Capacity score
SAGE-718	A Study to Evaluate the Safety and Tolerability of SAGE-718 in Participants With Huntington’s Disease	NCT05655520	Measure number and severity of TEAEs Measure number of participants that withdraw due to adverse events Measure change from baseline in clinical laboratory and electrocardiogram parameters Measure change in baseline in C-SSRS responses
RO7234292 (RG6042)	An Open-Label Extension Study to Evaluate Long-Term Safety and Tolerability of RO7234292 (RG6042) in Huntington’s Disease	NCT03842969	Measure number and severity of TEAEs Measure number of participants with suicidal ideation/behavior and self-injurious behavior without suicidal intent based on C-SSRS scale Measure change from baseline in MoCA

## Data Availability

Data sharing is not applicable.

## References

[1] Anil M., Mason S.L., Barker R.A. (2020). The clinical features and progression of late-onset versus younger-onset in an adult cohort of Huntington’s Disease patients. J. Huntington’s Dis..

[2] Ross C.A., Aylward E.H., Wild E.J., Langbehn D.R., Long J.D., Warner J.H., Scahill R.I., Leavitt B.R., Stout J.C., Paulsen J.S. (2014). Huntington disease: Natural history, biomarkers and prospects for therapeutics. Nat. Rev. Neurol..

[3] McColgan P., Tabrizi S.J. (2018). Huntington’s disease: A clinical review. Eur. J. Neurol..

[4] Rawlins M.D., Wexler N.S., Wexler A.R., Tabrizi S.J., Douglas I., Evans S.J.W., Smeeth L. (2016). The prevalence of Huntington’s disease. Neuroepidemiology.

[5] Rodríguez-Santana I., Mestre T., Squitieri F., Willock R., Arnesen A., Clarke A., D’Alessio B., Fisher A., Fuller R., Hamilton J.L. (2023). Economic burden of Huntington disease in Europe and the USA: Results from the Huntington’s Disease Burden of Illness study. Eur. J. Neurol..

[6] Shaw E., Mayer M., Ekwaru P., McMullen S., Graves E., Wu J.W., Budd N., Maturi B., Cowling T., Mestre T.A. (2022). Epidemiology and economic burden of Huntington’s disease: A Canadian provincial public health system perspective. J. Med. Econ..

[7] Myers R.H. (2004). Huntington’s disease genetics. NeuroRx.

[8] van der Zwaan K.F., Mentink M.D.C., Jacobs M., Roos R.A.C., de Bot S.T. (2022). Huntington’s disease influences employment before and during clinical manifestation: A systematic review. Park. Relat. Disord..

[9] Wright G.E.B., Black H.F., Collins J.A., Gall-Duncan T., Caron N.S., Pearson C.E., Hayden M.R. (2020). Interrupting sequence variants and age of onset in Huntington’s disease: Clinical implications and emerging therapies. Lancet Neurol..

[10] Li S.H., Schilling G., Young W.S., Li X.J., Margolis R.L., Stine O.C., Wagster M.V., Abbott M.H., Franz M.L., Ranen N.G. (1993). Huntington’s disease gene (IT15) is widely expressed in human and rat tissues. Neuron.

[11] Schulte J., Littleton J.T. (2011). The biological function of the Huntingtin protein and its relevance to Huntington’s Disease pathology. Curr. Trends Neurol..

[12] Saudou F., Humbert S. (2016). The biology of Huntingtin. Neuron.

[13] Hackam A.S., Hodgson J.G., Singaraja R., Zhang T., Gan L., Gutekunst C.A., Hersch S.M., Hayden M.R. (1999). Evidence for both the nucleus and cytoplasm as subcellular sites of pathogenesis in Huntington’s disease in cell culture and in transgenic mice expressing mutant huntingtin. Philos. Trans. R. Soc. Lond. B Biol. Sci..

[14] Davies S.W., Turmaine M., Cozens B.A., DiFiglia M., Sharp A.H., Ross C.A., Scherzinger E., Wanker E.E., Mangiarini L., Bates G.P. (1997). Formation of Neuronal Intranuclear Inclusions Underlies the Neurological Dysfunction in Mice Transgenic for the HD Mutation. Cell.

[15] Reiner A., Dragatsis I., Dietrich P. (2011). Genetics and Neuropathology of Huntington’s Disease. Int. Rev. Neurobiol..

[16] Almqvist E.W., Bloch M., Brinkman R., Craufurd D., Hayden M.R. (1999). A worldwide assessment of the frequency of suicide, suicide attempts, or psychiatric hospitalization after predictive testing for Huntington disease. Am. J. Hum. Genet..

[17] Petrella L.I., Castelhano J.M., Ribeiro M., Sereno J.V., Gonçalves S.I., Laço M.N., Hayden M.R., Rego A.C., Castelo-Branco M. (2018). A whole brain longitudinal study in the YAC128 mouse model of Huntington’s disease shows distinct trajectories of neurochemical, structural connectivity and volumetric changes. Hum. Mol. Genet..

[18] Blumenstock S., Dudanova I. (2020). Cortical and striatal circuits in Huntington’s disease. Front. Neurosci..

[19] Rocha G.S., Freire M.A.M., Britto A.M., Paiva K.M., Oliveira R.F., Fonseca I.A.T., Araújo D.P., Oliveira L.C., Guzen F.P., Morais P.L.A.G. (2023). Basal ganglia for beginners: The basic concepts you need to know and their role in movement control. Front. Syst. Neurosci..

[20] Zielonka D., Piotrowska I., Marcinkowski J.T., Mielcarek M. (2014). Skeletal muscle pathology in Huntington’s disease. Front. Physiol..

[21] Bonomo R., Elia A.E., Bonomo G., Romito L.M., Mariotti C., Devigili G., Cilia R., Giossi R., Eleopra R. (2021). Deep brain stimulation in Huntington’s disease: A literature review. Neurol. Sci..

[22] Bonelli R.M., Cummings J.L. (2008). Frontal-subcortical dementias. Neurologist.

[23] van Lonkhuizen P.J.C., Frank W., Heemskerk A.W., van Duijn E., de Bot S.T., Mühlbäck A., Landwehrmeyer G.B., Chavannes N.H., Meijer E., HEALTHE-RND Consortium (2023). Quality of life, health-related quality of life, and associated factors in Huntington’s disease: A systematic review. J. Neurol..

[24] Galyan S.M., Ewald C.Y., Jalencas X., Masrani S., Meral S., Mestres J. (2022). Fragment-based virtual screening identifies a first-in class preclinical drug for Huntington’s disease. Sci. Rep..

[25] Huntington’s Disease. https://www.mayoclinic.org/diseases-conditions/huntingtons-disease/diagnosis-treatment/drc-20356122.

[26] Estevez-Fraga C., Tabrizi S.J., Wild E.J. (2022). Huntington’s Disease clinical trials corner: November 2022. J. Huntington’s Dis..

[27] Blonde L. (2000). Management of type 2 diabetes: Update on new pharmacological options. Manag. Care.

[28] Kajbaf F., De Broe M.E., Lalau J.D. (2016). Therapeutic concentrations of metformin: A systematic review. Clin. Pharmacokinet..

[29] Song Y., Wu Z., Zhao P. (2022). The function of metformin in aging-related musculoskeletal disorders. Front. Pharmacol..

[30] Trujillo-Del Río C., Tortajada-Pérez J., Gómez-Escribano A.P., Casterá F., Peiró C., Millán J.M., Herrero M.J., Vázquez-Manrique R.P. (2022). Metformin to treat Huntington disease: A pleiotropic drug against a multi-system disorder. Mech. Ageing Dev..

[31] Faria J., Negalha G., Azevedo A., Martel F. (2019). Metformin and breast cancer: Molecular targets. J. Mammary Gland Biol. Neoplasia.

[32] Ronnett G.V., Ramamurthy S., Kleman A.M., Landree L.E., Aja S. (2009). AMPK in the brain: Its roles in energy balance and neuroprotection. J. Neurochem..

[33] Sanchis A., García-Gimeno M.A., Cañada-Martínez A.J., Sequedo M.D., Millán J.M., Sanz P., Vázquez-Manrique R.P. (2019). Metformin treatment reduces motor and neuropsychiatric phenotypes in the zQ175 mouse model of Huntington disease. Exp. Mol. Med..

[34] Hervás D., Fornés-Ferrer V., Gómez-Escribano A.P., Sequedo M.D., Peiró C., Millán J.M., Vázquez-Manrique R.P. (2017). Metformin intake associates with better cognitive function in patients with Huntington’s disease. PLoS ONE.

[35] Cai H., Cong W.N., Ji S., Rothman S., Maudsley S., Martin B. (2012). Metabolic dysfunction in Alzheimer’s disease and related neurodegenerative disorders. Curr. Alzheimer Res..

[36] Lange J., Gillham O., Flower M., Ging H., Eaton S., Kapadia S., Neueder A., Duchen M.R., Ferretti P., Tabrizi S.J. (2023). PolyQ length-dependent metabolic alterations and DNA damage drive human astrocyte dysfunction in Huntington’s disease. Prog. Neurobiol..

[37] Almikhlafi M.A., Karami M.M., Jana A., Alqurashi T.M., Majrashi M., Alghamdi B.S., Ashraf G.M. (2023). Mitochondrial medicine: A promising therapeutic option against various neurodegenerative disorders. Curr. Neuropharmacol..

[38] Solís-Maldonado M., Miró M.P., Acuña A.I., Covarrubias-Pinto A., Loaiza A., Mayorga G., Beltrán F.A., Cepeda C., Levine M.S., Concha I.I. (2018). Altered lactate metabolism in Huntington’s disease is dependent on GLUT3 expression. CNS Neurosci. Ther..

[39] Szablewski L. (2017). Glucose transporters in brain: In health and in Alzheimer’s disease. J. Alzheimer’s Dis..

[40] Chaves G., Stanley J., Pourmand N. (2019). Mutant huntingtin affects diabetes and Alzheimer’s markers in human and cell models of Huntington’s Disease. Cells.

[41] Andreassen O.A., Dedeoglu A., Stanojevic V., Hughes D.B., Browne S.E., Leech C.A., Ferrante R.J., Habener J.F., Beal M.F., Thomas M.K. (2002). Huntington’s disease of the endocrine pancreas: Insulin deficiency and diabetes mellitus due to impaired insulin gene expression. Neurobiol. Dis..

[42] Siesling S., van Vugt J.P., Zwinderman K.A., Kieburtz K., Roos R.A. (1998). Unified Huntington’s disease rating scale: A follow up. Mov. Disord..

[43] Gómez-Ansón B., Alegret M., Muñoz E., Sainz A., Monte G.C., Tolosa E. (2007). Decreased frontal choline and neuropsychological performance in preclinical Huntington disease. Neurology.

[44] Zilliox L.A., Chadrasekaran K., Kwan J.Y., Russell J.W. (2016). Diabetes and cognitive impairment. Curr. Diabetes Rep..

[45] (2010). FDA Center for Drug Evaluation and Research Medical Review Application Number: 021879Orig1s000. https://www.accessdata.fda.gov/drugsatfda_docs/nda/2010/021879Orig1s000MedR.pdf.

[46] Price D.D., Mao J., Frenk H., Mayer D.J. (1994). The N-methyl-D-asparate receptor antagonist dextromethorphan selectively reduces temporal summation of second pain in man. Pain.

[47] Patatanian E., Casselman J. (2014). Dextromethorphan/quinidine for the treatment of pseudobulbar affect. Consult. Pharm..

[48] Nabizadeh F., Nikfarjam M., Azami M., Sharifkazemi H., Sodeifian F. (2022). Pseudobulbar affect in neurodegenerative diseases: A systematic review and meta-analysis. J. Clin. Neurosci..

[49] Persaud A., Fares M.A., Giles S., Gaitour E., Shneyder N. (2018). Laughing and dancing: A case report of Pseudobulbar Affect in late onset Huntington’s Disease. Neurology.

[50] Scorr L.M., Factor S.A. (2018). VMAT2 inhibitors for the treatment of tardive dyskinesia. J. Neurol. Sci..

[51] Yaffe D., Forrest L.R., Schuldiner S. (2018). The ins and outs of vesicular monoamine transporters. J. Gen. Physiol..

[52] Harriott N.D., Williams J.P., Smith E.B., Bozigian H.P., Grigoriadis D.E. (2018). VMAT2 inhibitors and the path to Ingrezza (valbenazine). Prog. Med. Chem..

[53] Dean M., Sung V.W. (2018). Review of deutetrabenazine: A novel treatment for chorea associated with Huntington’s disease. Drug Des. Dev. Ther..

[54] Sung V.W., Iyer R.G., Gandhi S.K., Abler V., Davis B., Irwin D.E., Anderson K.E. (2018). Retrospective analysis of healthcare resource use, treatment patterns, and treatment-related events in patients with Huntington’s Disease-associated chorea initiated on tetrabenazine. J. Health Econ. Outcomes Res..

[55] Claassen D.O., Carroll B., De Boer L.M., Wu E., Ayyagari R., Gandhi S., Stamler D. (2017). Indirect tolerability comparison of deutetrabenazine and tetrabenazine for Huntington disease. J. Clin. Mov. Disord..

[56] https://www.drugs.com/history/austedo.html.

[57] Impact of Deutetrabenazine on Functional Speech and Gait Dynamics in Huntington Disease. https://classic.clinicaltrials.gov/ct2/results?cond=&term=Impact+of+Deutetrabenazine+on+Functional+Speech+and+Gait+Dynamics+in+Huntington+Disease&cntry=&state=&city=&dist=.

[58] Stipancic K.L., Tjaden K., Wilding G. (2016). Comparison of intelligibility measures for adults with Parkinson’s Disease, adults with Multiple Sclerosis and healthy control. J. Speech Lang. Hear. Res..

[59] Furr Stimming E., Claassen D.O., Kayson E., Goldstein J., Mehanna R., Zhang H., Liang G.S., Haubenberger D., Huntington Study Group KINECT-HD Collaborators (2023). Safety and efficacy of valbenazine for the treatment of chorea associated with Huntington’s disease (KINECT-HD): A phase 3, randomized, double-blind, placebo-controlled trial. Lancet Neurol..

[60] Szczakowska A., Gabryelska A., Gawlik-Kotelnicka O., Strzelecki D. (2023). Deep brain stimulation in the treatment of tardive dyskinesia. J. Clin. Med..

[61] https://www.drugs.com/history/ingrezza.html.

[62] Koch J., Shi W.X., Dashtipour K. (2020). VMAT2 inhibitors for the treatment of hyperkinetic movement disorders. Pharmacol. Ther..

[63] Frank S., Testa C., Edmondson M.C., Goldstein J., Kayson E., Leavitt B.R., Oakes D., O’Neill C., Vaughan C., Whaley J. (2022). The safety of deutetrabenazine for chorea in Huntington Disease: An open-label extension study. CNS Drugs.

[64] https://classic.clinicaltrials.gov/ct2/show/NCT04400331.

[65] Ferguson M.W., Kennedy C.J., Palpagama T.H., Waldvogel H.J., Faull R.L.M., Kwakowsky A. (2022). Current and possible future therapeutic options for Huntington’s Disease. J. Cent. Nerv. Syst. Dis..

[66] Song W.P., Jin L.Y., Zhu M.D., Wang H., Xia D.S. (2023). Clinical trials using dental stem cells: 2022 update. World J. Stem Cells.

[67] Ueda T., Inden M., Ito T., Kurita H., Hozumi I. (2020). Characteristics and therapeutic potential of dental pulp stem cells on neurodegenerative diseases. Front. Neurosci..

[68] Li B., Ouchi T., Cao Y., Zhao Z., Men Y. (2021). Dental-derived mesenchymal stem cells: State of the art. Front. Cell Dev. Biol..

[69] Wenceslau C.V., de Souza D.M., Mambelli-Lisboa N.C., Ynoue L.H., Araldi R.P., da Silva J.M., Pagani E., Haddad M.S., Kerkis I. (2022). Restoration of BDNF, DARPP32, and D2R expression following intravenous infusion of human immature dental pulp stem cells in Huntington’s Disease 3-NP rat model. Cells.

[70] Sramkó B., Földes A., Kádár K., Varga G., Zsembery Á., Pircs K. (2023). The wisdom in teeth: Neuronal differentiation of dental pulp cells. Cell Reprogram..

[71] Clinical Extension Study for Safety and Efficacy Evaluation of Cellavita-HD Administration in Huntington’s Patients. https://classic.clinicaltrials.gov/ct2/show/NCT04219241?term=Clinical+Extension+Study+for+Safety+and+Efficacy+Evaluation+of+Cellavita-HD+Administration+in+Huntington%E2%80%99s+Patients&draw=2&rank=1.

[72] Waters S., Tedroff J., Ponten H., Klamer D., Sonesson C., Waters N. (2018). Pridopidine: Overview of pharmacology and rationale for its use in Huntington’s Disease. J. Huntington’s Dis..

[73] Grachev I.D., Meyer P.M., Becker G.A., Bronzel M., Marsteller D., Pastino G., Voges O., Rabinovich L., Knebel H., Zientek F. (2021). Sigma-1 and dopamine D2/D3 receptor occupancy of pridopidine in healthy volunteers and patients with Huntington disease: A [^18^F] fluspidine and [^18^F] fallypride PET study. Eur. J. Nucl. Med. Mol. Imaging.

[74] Squitieri F., Di Pardo A., Favellato M., Amico E., Maglione V., Frati L. (2015). Pridopidine, a dopamine stabilizer, improves motor performance and shows neuroprotective effects in Huntington disease R6/2 mouse model. J. Cell. Mol. Med..

[75] What is Pridopidine?. https://www.Prilenia.com.

[76] Eddings C.R., Arbez N., Akimov S., Geva M., Hayden M.R., Ross C.A. (2019). Pridopidine protects neurons from mutant-huntingtin toxicity via the sigma-1 receptor. Neurobiol. Dis..

[77] Reilmann R., McGarry A., Grachev I.D., Savola J.M., Borowsky B., Eyal E., Gross N., Langbehn D., Schubert R., Wickenberg A.T. (2019). Safety and efficacy of pridopidine in patients with Huntington’s disease (PRIDE-HD): A phase 2, randomized, placebo-controlled, multicentre, dose-ranging study. Lancet Neurol..

[78] Tabrizi S.J., Estevez-Fraga C., van Roon-Mom W.M.C., Flower M.D., Scahill R.I., Wild E.J., Muñoz-Sanjuan I., Sampaio C., Rosser A.E., Leavitt B.R. (2022). Potential disease-modifying therapies for Huntington’s disease: Lessons learned and future opportunities. Lancet Neurol..

[79] https://clinicaltrials.gov/ct2/show/NCT04556656.

[80] Hill M.D., Blanco M.J., Salituro F.G., Bai Z., Beckley J.T., Ackley M.A., Dai J., Doherty J.J., Harrison B.L., Hoffmann E.C. (2022). SAGE-718: A first-in-class *N*-methyl-d-aspartate receptor positive allosteric modulator for the potential treatment of cognitive impairment. J. Med. Chem..

[81] Leoni V., Mariotti C., Tabrizi S.J., Valenza M., Wild E.J., Henley S.M., Hobbs N.Z., Mandelli M.L., Grisoli M., Björkhem I. (2008). Plasma 24S-hydroxycholesterol and caudate MRI in pre-manifest and early Huntington’s disease. Brain.

[82] Leoni V., Long J.D., Mills J.A., Di Donato S., Paulsen J.S., PREDICT-HD Study Group (2013). Plasma 24S-hydroxycholeteral correlation with markers of Huntington disease progression. Neurobiol. Dis..

[83] Byun S., Lee M., Kim M. (2022). Gene therapy for Huntington’s disease: The final strategy for a cure?. J. Mov. Disord..

[84] Rasul M.F., Hussen B.M., Salihi A., Ismael B.S., Jalal P.J., Zanichelli A., Jamali E., Baniahmad A., Ghafouri-Fard S., Basiri A. (2022). Strategies to overcome the main challenges of the use of CRISPR/Cas9 as a replacement for cancer therapy. Mol. Cancer.

[85] Scoles D.R., Pulst S.M. (2019). Antisense therapies for movement disorders. Mov. Disord..

[86] https://www.clinicaltrialsarena.com/comment/roche-tominersen-huntingtons-asset/?cf-view.

[87] Tabrizi S.J., Leavitt B.R., Landwehrmeyer G.B., Wild E.J., Saft C., Barker R.A., Blair N.F., Craufurd D., Priller J., Rickards H. (2019). Targeting huntingtin expression in patients with Huntington’s Disease. N. Engl. J. Med..

[88] Tang B.L. (2020). Could metformin be therapeutically useful in Huntington’s disease?. Rev. Neurosci..

[89] Russell R.D., Black L.J., Begley A. (2022). Nutrition education programs for adults with neurological diseases are lacking: A scoping review. Nutrients.

[90] Marder K., Zhao H., Eberly S., Tanner C.M., Oakes D., Shoulson I., Huntington Study Group (2009). Dietary intake in adults at risk for Huntington disease: Analysis of PHAROS research participants. Neurology.

[91] https://hdsa.org/find-help/living-well-with-hd/nutrition/.

[92] Kumar R.R., Singh L., Thakur A., Singh S., Kumar B. (2022). Role of vitamins in neurodegenerative diseases: A review. CNS Neurol. Disord. Drug Targets.

[93] https://hdsa.org/wp-content/uploads/2015/02/11695.pdf.

[94] Genetic Modifiers of Huntington’s Disease (GeM-HD) Consortium (2015). Identification of genetic factors that modify clinical onset of Huntington’s disease. Cell.

[95] Moss D.J.H., Pardiñas A.F., Langbehn D., Lo K., Leavitt B.R., Roos R., Durr A., Mead S., TRACK-HD Investigators, REGISTRY Investigators (2017). Identification of genetic variants associated with Huntington’s disease progression: A genome-wide association study. Lancet Neurol..

[96] Maiuri T., Suart C.E., Hung C.L.K., Graham K.J., Barba Bazan C.A., Truant R. (2019). DNA damage repair in Huntington’s Disease and other neurodegenerative diseases. Neurotherapeutics.

[97] Essa M.M., Moghadas M., Ba-Omar T., Walid Qoronfleh M., Guillemin G.J., Manivasagam T., Justin-Thenmozhi A., Ray B., Bhat A., Chidambaram S.B. (2019). Protective effects of antioxidants in Huntington’s Disease: An extensive review. Neurotox. Res..

[98] Gioia U., Francia S., Cabrini M., Brambillasca S., Michelini F., Jones-Weinert C.W., d’Adda di Fagagna F. (2019). Pharmacological boost of DNA damage response and repair by enhanced biogenesis of DNA damage response RNAs. Sci. Rep..

